# A Multi-Hop Cluster Routing Algorithm for Wireless Sensor Networks Targeting Narrow Space Monitoring

**DOI:** 10.3390/s26134127

**Published:** 2026-06-30

**Authors:** Jiawei Zhang, Jiguang Yang, Shannong Zheng, Jiuyuan Huo

**Affiliations:** 1 Faculty of Science and Technology, Beijing Normal-Hong Kong Baptist University, Zhuhai 519087, China; 2 School of Electronic and Information Engineering, Lanzhou Jiaotong University, Lanzhou 730070, China

**Keywords:** narrow and long space, WSN, sparrow search algorithm, clustering routing algorithm, non-uniform clustering

## Abstract

Wireless sensor networks (WSNs) are recognized as a promising enabling technology for health monitoring of elongated infrastructures such as bridges, tunnels and railways. However, the significant distribution span of WSN nodes within narrow spaces requires monitoring data to be transmitted to the base station via multi-hop routing, which poses higher demands on network energy efficiency and lifespan. This paper proposes a Multi-hop Cluster Routing Algorithm based on an Improved Sparrow Search Algorithm (ISSAMC) aimed at optimizing the optimal multi-hop path from cluster heads (CHs) to the base station, thereby extending the stability period and overall lifespan of WSNs in narrow spaces. The ISSAMC first employs a non-uniform clustering mechanism, taking into account the residual energy of nodes and the distance to the base station, to generate a CH distribution that aligns with the topological characteristics of the narrow structure. Next, a multi-objective fitness function is constructed to simultaneously minimize the total energy consumption of the CHs and the variance in energy consumption, along with a dynamic weight adjustment strategy to adapt to the time-varying characteristics of the network state. Finally, multi-hop path optimization is performed using an improved SSA that incorporates strategies such as population initialization based on Sobol sequences, discrete encoding and decoding mechanisms, and crossover techniques, resulting in high-quality multi-hop paths. Simulation results show that under the unified ideal simulation benchmark, compared with MH-LEACH, GAECH, BEBMCR and EBPSO algorithms, ISSAMC improves the network stability period by 231%, 94%, 60% and 49.5%, respectively, and extends the overall network lifetime by 55%, 31%, 25.5% and 24%, respectively.

## 1. Introduction

Wireless sensor networks (WSNs) are regarded as a promising enabling technology in the field of Structural Health Monitoring (SHM) due to their low cost, flexible deployment, and strong scalability. They have shown broad application prospects in elongated infrastructure scenarios such as bridges, tunnels, railways, and pipelines, where they can be deployed to collect physical parameters including vibration, strain, temperature, and humidity [1,2]. Unlike the square planar monitoring areas in traditional studies, narrow spaces impose unique constraints on WSN communications: sensor nodes are typically linearly distributed along the length, while the width is extremely limited. This topological characteristic necessitates that the monitoring data be transmitted through multi-hop paths to a static base station (BS), which is usually located at one end of the network [3].

In such environments, energy efficiency is one of the primary challenges. Sensor nodes rely on battery power and must operate continuously for months or even years without battery replacement. Cluster-based multi-hop routing algorithms are widely used to reduce energy consumption because they can perform data aggregation at the cluster head (CH) and forward data to the BS using multi-hop transmission [4,5]. However, traditional clustering methods (such as LEACH and its variants) are primarily designed for square, high-density networks and implicitly assume that nodes can communicate with the base station over short distances. When these algorithms are directly applied to elongated topologies, they reveal the following three main limitations:

1. Uneven CH Distribution: The difference in distance from CHs to the BS generated by uniform clustering mechanisms is significant, causing nodes located further from the BS to deplete their energy prematurely due to long-distance transmission.
2. Ineffective Inter-cluster Routing: Many algorithms employ greedy or single-criteria approaches (e.g., nearest neighbor) to select the next hop CH. While this may minimize single-hop energy consumption, it fails to balance the total energy consumption across the entire link, leading to the formation of bottleneck nodes that can die prematurely.
3. Static Optimization Ignoring Time-Varying Network Characteristics: As node energy is consumed, the network state continuously changes. The multi-hop path selection strategies of most algorithms do not adjust over time, resulting in a severe decline in performance during the later stages of network operation.

To overcome the above limitations, researchers have attempted to integrate various optimization algorithms, such as Genetic Algorithm (GA) [6], Particle Swarm Optimization (PSO) [7], and Ant Colony Optimization (ACO) [8], into clustering and routing decisions. In recent years, the Sparrow Search Algorithm (SSA) [9,10] has attracted attention due to its fast convergence and strong global search capability. However, when applied to discrete combinatorial optimization problems such as multi-hop path selection in WSNs, the standard SSA tends to suffer from premature convergence and loss of population diversity. Moreover, most existing SSA-based routing schemes take only residual energy and distance from nodes to the base station as optimization objectives, neglecting load balance among CHs, a critical factor for prolonging network lifetime in elongated areas.

To address the above issues, this paper proposes an Improved SSA-based Multi-hop Clustering algorithm (ISSAMC) specifically designed for monitoring scenarios in elongated spaces. The main contributions of this paper are as follows:

1. Based on the concept of non-uniform clustering, a formula for calculating the node competition radius is proposed by comprehensively considering the residual energy of nodes and their distance to the sink node, thereby achieving a more rational distribution of CHs.
2. A novel fitness function is designed, and a nonlinear dynamic weighting mechanism based on the network residual energy threshold is introduced to enable the objective function to adapt to the time-varying characteristics of the network.
3. Aiming at the applicability of the algorithm to elongated spaces and its global search capability, targeted improvements are made to the SSA, and the improved algorithm is used to search for the optimal inter-cluster multi-hop path.
4. The proposed ISSAMC algorithm can effectively meet the practical application requirements of monitoring scenarios in elongated spaces.

Simulation results demonstrate that under the unified simulation benchmark of the elongated scenario, compared with the four representative algorithms MH-LEACH, GAECH, BEBMCR and EBPSO, ISSAMC increases the network stability period by 231%, 94%, 60% and 49.5%, respectively, and prolongs the total network lifetime by 55%, 31%, 25.5% and 24%, respectively. The above results verify the performance advantage of the proposed algorithm under idealized simulation settings and can provide theoretical reference for the routing design of wireless sensing monitoring systems for elongated infrastructures such as bridges, tunnels, railways and pipelines. As indicated by Paterova et al. [11] in their study on energy consumption characteristics of low-power wireless sensing nodes, the energy consumption of sensing acquisition, MCU processing and sleep standby accounts for a significantly lower proportion than that of wireless communication. Accordingly, the model in this study focuses on the comparative analysis of communication energy consumption at the routing layer and does not incorporate engineering details such as link retransmission and fine-grained synchronization for the time being; the relevant conclusions mainly support the relative performance comparison of routing algorithms, and further targeted verification is required for practical engineering deployment.

The remainder of this paper is organized as follows: Section 2 reviews and analyzes related literature; Section 3 presents the network model and basic algorithms; Section 4 describes the proposed ISSAMC algorithm in detail; Section 5 provides simulation results and analysis; and Section 6 concludes the paper and discusses future work.

## 2. Related Work

Clustering routing algorithms extend the network lifetime by partitioning the network into clusters and electing CHs to aggregate data and balance energy consumption. The LEACH algorithm [12] is a classic clustering routing algorithm. However, due to the random generation of CHs and direct communication between CHs and the BS, nodes consume a significant amount of energy in a short period. Consequently, LEACH is not suitable for monitoring elongated spaces where data transmission distances are large. As an important improvement of LEACH, PEGASIS [13] optimizes energy consumption through a chain-based structure. Nevertheless, a single node failure in the chain may cause the entire link to fail, and data must be transmitted over multiple hops to reach the CH, leading to increased communication delay. Neto et al. [14] introduced a multi-hop mechanism into LEACH and proposed the MH-LEACH routing algorithm, in which CHs forward data to their nearest CH to shorten transmission distances and reduce energy consumption. However, the constructed multi-hop paths contain many critical nodes, resulting in poor robustness. Al-Sodairi et al. [15] proposed an enhanced multi-hop LEACH algorithm to prolong network lifetime, which considers both the residual energy of nodes and the number of times a node has acted as CH when calculating the probability of an ordinary node becoming a CH. Jin et al. [16] proposed a centralized multi-hop routing based on a multi-start minimum spanning forest (LEACH-CMF). Building on LEACH, they introduced a minimum spanning tree algorithm to select relay nodes with the lowest relay cost and generate appropriate multi-hop paths. Although adding a multi-hop mechanism to LEACH can effectively extend network lifetime, the CH election process does not control the cluster size, making it difficult to establish a reasonable cluster structure and effectively mitigate the “hot spot” problem [17].

Elkamel et al. [18] conducted an in-depth investigation into the “hot spot” problem in multi-hop routing and pointed out that non-uniform clustering can effectively mitigate this issue. Li Chengfa et al. [19] proposed a novel multi-hop routing algorithm based on non-uniform clustering, in which the cluster size is positively correlated with the distance from the CH to the BS—i.e., clusters near the BS are small, while those far from the sink node are large. He et al. [20] proposed a low-energy non-uniform clustering routing algorithm that takes quality of service into account. The algorithm designs a node competition radius calculation formula that comprehensively considers the distance from the CH to the sink node and the artificial cost. Moreover, CH nodes use a greedy algorithm to construct multi-hop paths among clusters. Nivedhitha et al. [21] proposed a dynamic multi-hop energy-efficient routing protocol (DMEERP) to balance network reliability and energy consumption. The algorithm introduces a super CH (SCH) to address network disruption caused by the failure of certain CHs. Jing et al. [22] proposed a deterministic route search algorithm to enhance the reliability of data transmission in WSNs. By analyzing the relationship between hop count and delay, the method simplifies the search set for multi-hop paths among nodes and determines the inter-cluster multi-hop path with the objective of minimizing delay. Mansi Gupta et al. [23] proposed an energy-efficient game-theoretic multi-hop routing algorithm (EGCR). The algorithm determines the optimal route by considering various factors such as node centrality, distance from the node to the BS, and residual energy.

The process of determining multi-hop routing paths among nodes in WSNs can be defined as a non-deterministic polynomial-time (NP) problem [24]. Heuristic optimization algorithms provide effective solutions to such problems, and consequently, the use of heuristic intelligent optimization algorithms to solve multi-objective optimization problems in WSN key technologies has become a research hotspot [25].

Ataul Bari et al. [26] proposed a GA-based energy-efficient routing scheme for two-tier sensor networks. In this method, chromosomes are encoded using integer coding, where the length of a chromosome equals the total number of CHs in the network, and the value of each gene represents the index of the next-hop CH. Zhang Rongbo et al. [27] adopted an ant colony optimization algorithm on the basis of EEUC to construct multi-hop paths among CHs, and experimental results showed that the proposed method outperformed the original EEUC. Baranidharan et al. [28] proposed a GA-based energy-efficient clustering hierarchy (GAECH) algorithm to prolong the network stability period and lifetime, where the total energy consumption of CHs and the variance of CH energy consumption are key components of the fitness function. Mohammed Al-Shalabi et al. [29] proposed a multi-hop routing algorithm based on an improved GA. In designing the fitness function, parameters such as the average distance from CHs to the BS are considered to reduce energy consumption during data transmission. Furthermore, a filtering operation is introduced during individual encoding to select high-quality CHs for constructing multi-hop paths, thereby improving algorithm efficiency. Aljapur Vinitha et al. [30] proposed a cat-salp swarm algorithm (C-SSA)-based energy-efficient routing protocol for WSNs, designing an objective function that comprehensively considers energy, delay, inter-cluster distance, and intra-cluster distance. Yu Xiuwu et al. [31] proposed a particle swarm optimization-based multi-hop routing algorithm to prolong network lifetime, comprehensively considering the distance from CHs to the BS and the residual energy of CHs when selecting relay nodes. K. Pushpa Rani et al. [32] proposed a fault-tolerant cluster-based routing technique to enhance network fault tolerance, adopting a battle royale optimization algorithm to select backup CHs (BKCHs) for improved fault tolerance and an improved particle swarm optimization algorithm to determine the optimal relay nodes for CHs. Marjan Kaedi et al. [33] proposed a two-level GA-based routing algorithm to simultaneously address CH selection and optimal multi-hop routing. The first-level GA selects CHs, while the second-level GA considers multi-hop routing among CHs. Yulin Gong et al. [34] designed an energy-balanced multi-hop routing algorithm for large-scale water quality monitoring, controlling the overlap ratio during CH election to avoid excessive overlap among CHs and employing the GA to search for optimal inter-cluster paths in parallel. X Luo et al. [35] focus on wireless sensor networks with energy-harvesting capabilities, aiming to reduce deployment cost and optimize random relay mechanisms under a fixed clustering architecture. X. Yang et al. [36] proposed an adaptive clustering routing algorithm, namely ACHB. Its core idea is to employ acoustic waves for omnidirectional intra-cluster data collection and optical waves for high-rate inter-cluster data transmission. Meanwhile, the algorithm improves cluster head reliability through a dynamic hierarchical cluster head backup mechanism, balances energy consumption using an adaptive clustering strategy based on node density and depth, and addresses the routing void problem through a backup-node-based mechanism.

Although the above multi-hop routing algorithms can effectively prolong the network lifetime in their respective application scenarios, they generally fail to adequately address the short stability period caused by unbalanced energy consumption among nodes. Moreover, these algorithms are not readily applicable to monitoring scenarios in elongated spaces. Given the special characteristics of elongated topologies, it is necessary not only to focus on energy balance among nodes in such WSNs but also to pay particular attention to the adaptability of the algorithm to the elongated spatial topology.

## 3. Theories

### 3.1. Energy Consumption Model

The energy consumption of sensor nodes primarily consists of data sensing, data processing, and data transmission, among which the energy consumed by data transmission is far greater than that of the other two parts. This paper adopts the energy model described in the LEACH algorithm [12]. The first-order radio energy consumption model adopted in this study follows the classical benchmark parameter system widely used in the field of WSN clustering routing. The core parameters, including the transmitting circuit power consumption, receiving circuit power consumption, and channel power amplification coefficients, are fully consistent with the settings in the original LEACH study and are also compatible with the typical datasheet values of mainstream low-power RF chips under the IEEE 802.15.4 standard [37]. The energy consumed by a sensor node to transmit k bits of data to a node at a distance of d meters, denoted as $E_{Tx}\left(k,d\right)$, is given by

(1) $$E_{Tx}\left(k,d\right)=\{\begin{array}{l} kE_{elec}+kE_{fs}d^{2},d<d_{0}\\ kE_{elec}+kE_{mp}d^{4},d\geq d_{0} \end{array}$$

The energy consumed by a node to receive k bits of data, denoted as $E_{Rx}\left(k\right)$, is given by

(2) $$E_{Rx}\left(k\right)=kE_{elec}$$

where $E_{elec}$ is the energy consumed by the transmitting and receiving circuits to process one bit of data; $E_{fs}$ is the amplifier power consumption under the free-space channel model; $E_{mp}$ is the amplifier power consumption under the multipath fading model; and $d_{0}$ is the distance threshold. When the transmission distance is less than $d_{0}$, the free-space channel model is adopted; otherwise, the multipath fading model is used. The value $d_{0}$ is obtained by Equation (3).

(3) $$d_{0}=\sqrt{\frac{E_{fs}}{E_{mp}}}$$

### 3.2. Network Model

**Figure 1:**
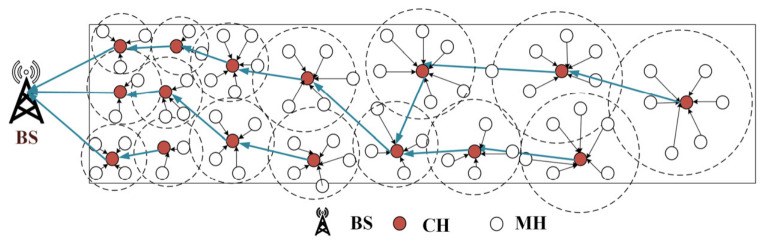
Network topology structure.

As shown in Figure 1, N nodes of the WSN are deployed in an elongated monitoring area of size D×L, where D is the width of the monitoring area, L is the length, and L≫D. Many scholars have conducted in-depth research on node deployment in such ribbon-shaped networks and have proposed several effective deployment schemes. The node deployment scheme adopted in this paper is the same as that in [38]. First, the entire ribbon-shaped network area of length L is divided into n sub-areas of equal distance, and the number of nodes in each sub-area is denoted as $N_{i}$. The node deployment formula is shown in Equation (4). The core logic of this deployment formula is to match the differences in multi-hop energy consumption under a linear topology. Therefore, it is applicable to long and narrow monitoring regions with a high aspect ratio, where the length is much greater than the width.

(4) $$N_{i}=\left[1+\frac{\left(2n-2i\right)E_{elec}+(n-i)E_{mp}d^{4}}{3E_{elec}+2E_{mp}d^{4}}\right]N$$

Since the number of nodes in each sub-area must be an integer, $N_{i}$ needs to be rounded off. The expression for calculating the total number of nodes in the *i*-th sub-area after rounding, denoted as $N_{i}$, is shown in Equation (5), where n is the total number of partitions of the entire network, and i is the sub-area index.

(5) $$N_{i}=\frac{\left[13+8\left(n-i\right)\right]}{4n^{2}+9n}N$$

The working principle of the ISSAMC algorithm is as follows: sensor nodes deployed at specific locations select CHs and multi-hop transmission paths according to the working mechanism. Ordinary nodes within a cluster send data to their CH, and the CH transmits the data to the BS via multi-hop routing. The entire network performs periodic data collection and transmission.

In addition, the following assumptions are made:

(1) All ordinary wireless sensor nodes in the network are homogeneous, battery-powered, and have the same initial energy. Once deployed, the positions of ordinary sensor nodes are immovable.
(2) The BS is located at one end of the monitoring area, fixed in position, with strong computing capability and unlimited energy.
(3) Each sensor node has a unique identity (ID) and communication address (Ad).
(4) Sensor nodes can appropriately adjust their transmission power according to the data transmission distance to reduce energy consumption.

The communication and energy consumption models in this study are established based on IEEE 802.15.4 (ZigBee), a short-range and low-power communication technology. Core parameters, including the node communication radius, transmitting and receiving power consumption, and transmission rate, are consistent with the typical values of this technology. Moreover, the intra-cluster TDMA time-slot scheduling mechanism is logically consistent with the slotted channel access mechanism of this standard.

### 3.3. Sparrow Search Algorithm

The Sparrow Search Algorithm (SSA) is a novel meta-heuristic algorithm first proposed in 2020 [9]. SSA updates individual positions by simulating the foraging and anti-predation behaviors of sparrows. Compared with traditional algorithms, SSA has the characteristics of a simple structure and ease of implementation, while requiring only a few control parameters and exhibiting good local search capability.

The Sparrow Search Algorithm divides individuals into producers, scroungers, and vigilantes. Among them, producers have higher energy reserves, and their main task is to find areas rich in food sources and provide directions to scroungers. Scroungers choose to follow a producer to the area it has discovered for foraging. The role of the vigilantes is to monitor the rich food source areas discovered by the producers, preventing the population from being attacked by predators. The entire Sparrow Search Algorithm actually discovers the optimal solution in the solution space by continuously updating the positions of producers, scroungers, and vigilantes.

Typically, the top 10% or 20% of the population with the highest energy are set as producers, and their position update formula is shown in Equation (6).

(6) $$X_{i}^{t+1}=\{\begin{array}{ll} X_{i}^{t}\times exp\left(\frac{-i}{\sigma \times {iter}_{max}}\right), & R2<ST\\ X_{i}^{t}+Q\times L, & otherwise \end{array}$$

where $X_{i}^{t+1}$ denotes the position of individual $X_{i}$ at generation t+1, $X_{i}^{t}$ is its position at generation t, σ is a random number in the range [0, 1], ${iter}_{max}$ is the maximum number of iterations, Q is a random number following a normal distribution, and L is a 1×d unit vector. R2 and ST represent the alarm value and the safety threshold, respectively. When the alarm value is less than the safety threshold, it indicates that no predator has been detected in the current foraging area, allowing the producers to search over a broader region. Otherwise, all individuals in the population leave the current foraging area and move to other safe areas to forage.

The position update formula for scroungers is shown in Equation (7).

(7) $$X_{i}^{t+1}=\{\begin{array}{l} Q\times exp\left(\frac{X_{worst}^{t}-X_{i}^{t}}{i^{2}}\right),\;\;\;\;\;\;\;\;\;\;\;\;\;\;\;\;i>\frac{n}{2}\\ X_{best}^{t+1}+\left|X_{i}^{t}-X_{best}^{t+1}\right|\times A^{+}\times L,\;\;\;\;otherwise \end{array}$$

where $X_{best}^{t+1}$ represents the position of the fittest individual in generation t+1, and $X_{worst}^{t}$ represents the position of the individual with the worst fitness in generation t. A denotes a 1×d matrix whose elements are either 1 or −1, and $A^{+}=A^{T}{\left(AA^{T}\right)}^{-1}$.

The position update formula for vigilantes is shown in Equation (8).

(8) $$X_{i}^{t+1}=\{\begin{array}{ll} X_{best}^{t}+\rho \times \left|X_{i}^{t}-X_{best}^{t}\right|, & f_{i}>f_{g}\\ X_{i}^{t}+\varphi \times \left(\frac{X_{i}^{t}-X_{worst}^{t}}{\left(f_{i}-f_{worst}\right)+\varepsilon }\right), & f_{i}=f_{g} \end{array}$$

where $f_{i}$ represents the fitness value of the current individual i, $f_{worst}$ represents the worst fitness of the population, and $f_{g}$ represents the best fitness of the population. ρ is a random number between 0 and 1 used to control the step size of position updates. φ controls the direction of position updates and ranges from −1 to 1. ε is an infinitesimal quantity used to prevent the denominator $f_{i}-f_{worst}$ from becoming 0.

## 4. The Proposed Algorithm

The ISSAMC algorithm mainly consists of three phases: the preparation phase, the setup phase, and the data transmission phase. The main task of the preparation phase is for the BS to collect node information in the network and model the network. The setup phase is the core of the proposed algorithm, primarily accomplishing two tasks: CH selection and route establishment. The data phase is responsible for transmitting the data collected by nodes to the BS. The proposed algorithm is described in detail below.

### 4.1. Preparation Phase

The BS broadcasts an initialization message (Init_Msg) to the entire network, requiring each node to send its own information to the central node. Upon receiving the Init_Msg signal, each node packages its information, including location, energy status, node ID, etc., into a data packet (Node_Msg) and transmits it to the BS via multi-hop routing. The BS stores this information and updates the node information. ISSAMC is a centralized algorithm; both CH selection and inter-cluster route establishment are performed at the BS, thereby effectively conserving node energy and prolonging the network lifetime.

### 4.2. Clustering Based on Competitive Radius

Multi-hop transmission leads to a severe energy imbalance problem throughout the network. Numerous studies have shown that controlling the cluster size can, to some extent, mitigate the “energy hole” problem [39,40,41]. In this paper, we propose a novel node competition radius metric and determine the CHs in the network using a competition radius-based clustering algorithm. To improve the efficiency of CH selection, we first construct a candidate CH (CCH) set. According to the principle of regional average energy, nodes whose residual energy is not less than the regional average energy are added to the CCH set. The calculation formula for the regional average energy is shown in Equation (9).

(9) $$E_{av\_i}=\frac{\sum_{j\in N_{i}}E_{j}}{alive\_i},\;\;j\in N_{i}$$

where $N_{i}$ represents the set of currently alive nodes in region *i*, $E_{j}$ denotes the residual energy of node j, and alive_i indicates the total number of alive nodes in region i.

However, due to the large span of WSNs in elongated scenarios, the influence of the distance from a node to the BS on the cluster size is taken into account when designing the competition radius. Furthermore, a minimum competition radius $R_{min}$ is set to prevent excessive CHs in the area close to the BS, which would otherwise lead to energy imbalance among nodes. The specific calculation method of the node competition radius is shown in Equation (10).

(10) $$R_{C}\left(l\right)=\{\begin{array}{l} \left(1-\alpha \left(\frac{d_{maxBS}-d\left(l,BS\right)}{d_{maxBS}-d_{minBS}}\right)-\beta \left(\frac{E_{max}\left(i\right)-E_{l}}{E_{max}\left(i\right)}\right)\right)\times R_{max},R_{C}\left(l\right)>R_{min}\\ R_{min},\;otherwise \end{array}$$

where $R_{C}\left(l\right)$ denotes the competition radius of node l, $d_{maxBS}\left(i\right)$ is the maximum distance from any node in the network to the BS, $d_{minBS}\left(i\right)$ is the minimum distance from a node to the BS, d(l,BS) is the distance from node l to the sink node, $E_{max}\left(i\right)$ is the maximum residual energy among nodes in the *i*-th subnetwork, and $E_{l}$ represents the current residual energy of node l. *α* and *β* are used to control the influence of the distance metric and the energy metric on the cluster competition radius, respectively, satisfying α+β=1. When α increases, the influence of the node to BS distance on the competition radius is strengthened, resulting in a smaller competition radius for nodes closer to the BS and a more pronounced non-uniform clustering effect; when β increases, the influence of residual energy on the competition radius is strengthened, so nodes with higher residual energy obtain a larger competition radius and can undertake more intra-cluster management tasks. $R_{min}$ denotes the lower bound of the node competition radius, which is used to prevent excessive shrinkage of the competition radius near the base station and avoid overly dense cluster-head distribution that would increase management and control overhead. Considering the 50 m width constraint of the long and narrow region, this parameter is determined as 20 m through comparative simulations with multiple candidate values, thereby ensuring both complete regional coverage and balanced network energy consumption.

### 4.3. Construction of Multi-Hop Paths

Determining the multi-hop routing between CHs and the BS in an elongated WSN is a relatively complex problem. When the network scale is large and the topology is complex, finding the global optimum is very difficult and time-consuming. In recent years, many researchers have applied heuristic optimization algorithms to inter-cluster multi-hop routing optimization and achieved promising results [30]. The SSA is simple to implement and effective, making it suitable for solving many combinatorial optimization problems. However, the traditional SSA is not well-suited for discrete optimization problems [42]. To apply SSA to the inter-cluster path optimization problem, this paper enhances the traditional SSA with a Sobol sequence-based initialization method, discrete encoding and decoding mechanisms, and a crossover mechanism to determine the optimal multi-hop path.

#### 4.3.1. Encoding and Decoding of Solutions

**Figure 2:**
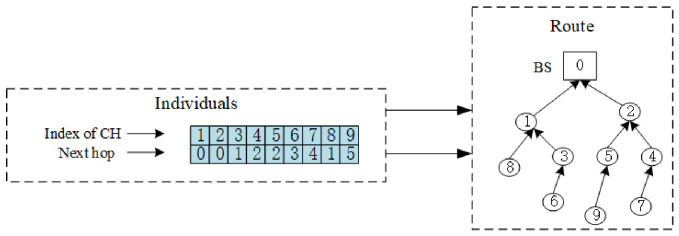
Sparrow individual and its corresponding route.

In ISSAMC, each sparrow individual represents an inter-cluster route, and the length of each sparrow individual is equal to the number of CHs. To more intuitively map the path to the actual network topology, this paper adopts an integer sequence for encoding sparrow individuals, where each element in the individual denotes the index of a CH on the path. Figure 2 illustrates a sparrow individual based on integer encoding and its corresponding route, from which the path from any CH to the BS can be determined. For example, the path from CH_8_ to the BS is CH_8_ → CH_1_ → BS, and the path from CH_6_ to the BS is CH_6_ → CH_3_ → CH_1_ → BS.

**Figure 3:**
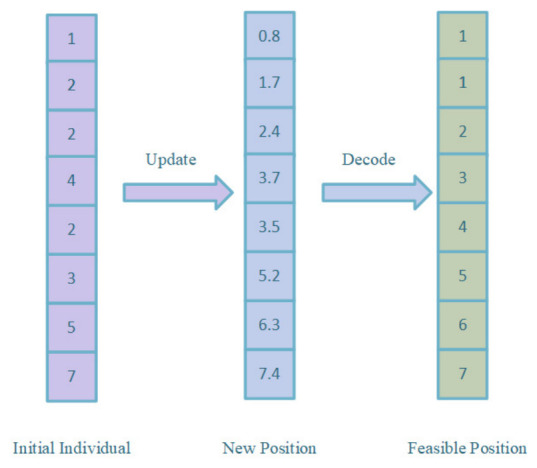
Decoding process of a sparrow individual.

Although encoding individuals as integer sequences can intuitively map the actual routes, individuals need to update their positions in a continuous solution space. During the update process, the solutions may contain decimals, leading to a large number of infeasible solutions [43]. To address this issue, this paper proposes a decoding method based on stochastic rounding. This method introduces randomness to each positional element and determines the rounding mode based on the generated random numbers. The decoding process is illustrated in Figure 3. The mathematical expression of the proposed decoding method is given in Equation (11).

(11) $$\mathrm{new\_X}\left(i,j\right)=\{\begin{array}{l} ceil\left(\mathrm{pre\_X}(i,j)\right),\;rand\geq p_{1}\\ floor\left(\mathrm{pre\_X}(i,j)\right),\;rand<p_{1} \end{array}$$

where pre_X(i,j) denotes the value of the *j*-th element of the *i*-th individual, ceil and floor represent the ceiling and floor operations, respectively, rand is a random number, and $p_{1}$ is a hyperparameter used to determine the rounding mode.

#### 4.3.2. Individual Initialization

**Figure 4:**
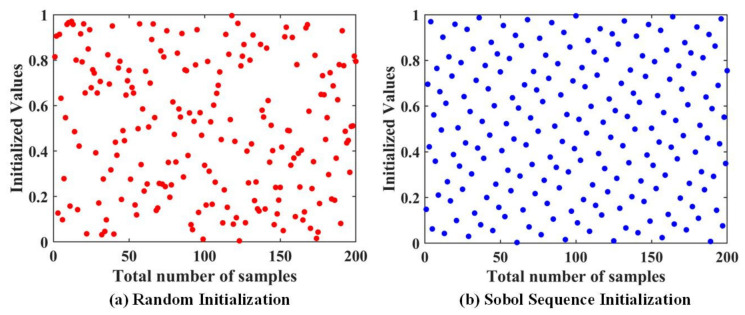
Comparison of Sobol sequence initialization and random initialization.

SSA uses pseudo-random numbers for individual initialization. However, the distribution of individuals generated by random initialization in the solution space is not sufficiently stable, which can easily lead to a large number of individuals concentrated in a limited subspace, thereby significantly affecting the algorithm’s performance. To address this issue, this paper proposes an individual initialization method based on the Sobol sequence. The Sobol sequence is a low-discrepancy sequence, meaning that the differences among sample points are relatively small. This results in a more uniform distribution of samples in the search space, helping to avoid getting trapped in local optimal solutions [44]. Figure 4 shows the distribution of 200 sample points using random initialization (Figure 4a) and Sobol sequence-based initialization (Figure 4b). It is evident that the nodes initialized with the Sobol sequence are more uniformly distributed. The mathematical expression of the proposed individual initialization is given in Equation (12).

(12) $$X_{n}=X_{min}+K_{n}\times \left(X_{max}-X_{min}\right)$$

where $X_{n}$ represents the initial position sequence of the *n*-th sparrow individual, $X_{min}$ and $X_{max}$ denote the lower and upper bounds of the search space, respectively, and $K_{n}$ represents the value of the *n*-th element in the generated Sobol sequence.

It should be noted that although the multi-hop path optimization problem in this study is essentially a discrete combinatorial problem, the algorithm adopts a solution framework based on continuous-space search and discrete encoding–decoding mapping. The uniform distribution property of the initial population in the continuous space can be transferred to the discrete feasible solution space. Therefore, Sobol sequence-based initialization can reduce the randomness of population distribution caused by random sampling and improve the stability of algorithm optimization.

#### 4.3.3. Lateral Crossover and Vertical Crossover Mechanisms

Although SSA exhibits excellent convergence speed, it tends to fall into local optima in the later stages of operation, resulting in low solution accuracy. To address this issue, this paper incorporates genetic optimization algorithms to improve the position update mechanism of the scroungers. By performing a lateral crossover operation on individuals, the solution space of the multidimensional problem is partitioned into semigroup hypercubes, enabling edge searches of the space. This approach reduces blind spots and enhances global optimization capability. Through vertical crossover, crossover operations are performed across different dimensions within the population, facilitating dimensions that have become stagnant in escaping local optima while maintaining the stability of other dimensions. Finally, the best individual positions after both crossover operations are retained, thereby achieving efficient solution of the algorithm.

The lateral crossover operation is inspired by the crossover operation in genetic algorithms, where different individuals in the population perform crossover at the same position. Its specific calculation formula is given in Equation (13).

(13) $$NX_{i,m}^{t+1}=r_{1}\times X_{i,m}^{t}+\left(1-r_{1}\right)\times X_{j,m}^{t}+c_{1}\times \left(X_{i,m}^{t}-X_{j,m}^{t}\right)$$

where $NX_{i,m}^{t+1}$ denotes the new value generated by the crossover operation between individuals $X_{i}^{t}$ and $X_{j}^{t}$ in the *m*-th dimension. Both $r_{1}$ and $c_{1}$ are hyperparameters, with $r_{1}$ ranging in [0, 1] and $c_{1}$ ranging in [−1, 1]. The individuals generated by lateral crossover need to be compared with the original individuals, and the better ones are retained.

The vertical crossover is inspired by the mutation operation in genetic algorithms, which enables the algorithm to achieve a better ability to escape local optima and improve solution accuracy [45]. The vertical crossover operation refers to performing crossover between elements of different dimensions within an individual to generate a new individual, as specifically expressed in Equation (14).

(14) $$NX_{i,m}^{t+1}=r_{2}\times X_{i,m}^{t}+\left(1-r_{2}\right)\times X_{i,z}^{t}$$

where $NX_{i,m}^{t+1}$ is the new value generated by performing the crossover operation between the element in the *m*-th dimension and the element in the *z*-th dimension of $X_{i}^{t}$, and $r_{2}$ is a hyperparameter with a value in [0, 1].

**Figure 5:**
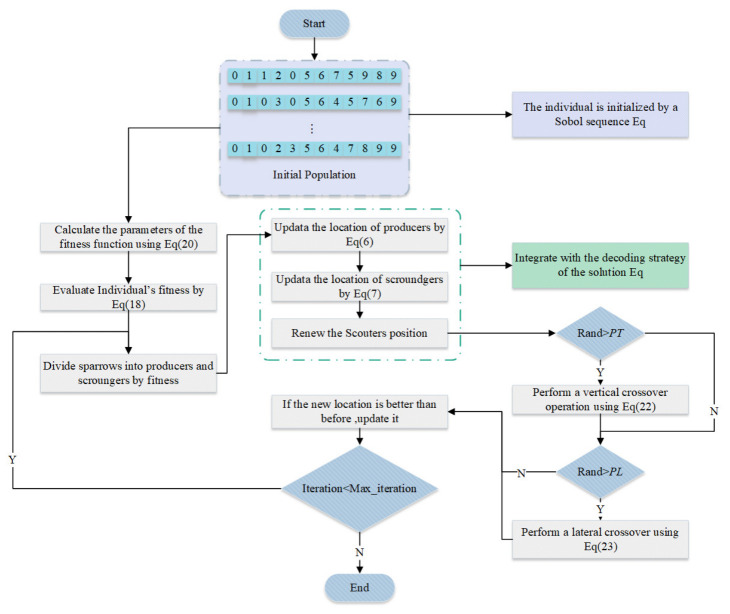
Flowchart of multi-hop path construction in ISSAMC.

In summary, the flowchart for finding the optimal inter-cluster multi-hop path in ISSAMC is shown in Figure 5.

#### 4.3.4. Design of the Objective Function

The lifespan of a WSN is influenced by many factors. When using heuristic algorithms to optimize its multi-hop routing, multiple optimization objectives are often considered, such as minimizing energy consumption, maximizing network lifetime, and maximizing the data transmission success rate. The fitness function needs to map these objectives appropriately to ensure that the algorithm advances toward a reasonable solution space during the search. In this paper, prolonging network lifetime and balancing energy consumption among nodes are taken as the optimization objective functions, which are quantitatively analyzed through the total energy consumption function and the energy consumption variance function. The definitions of the total energy consumption function and the energy consumption variance function are as follows:

- Total Energy Consumption Function

The total energy consumption is taken as part of the fitness function to holistically account for the energy consumption of the network. By minimizing the total energy consumption, the overall network lifetime can be effectively prolonged. The total energy consumption function is defined as the total energy consumed by all CHs (CHs) during data transmission, and its calculation is shown in Equation (15).

(15) $$f_{1}=\sum_{l\in CH}E_{cost}\left(l\right)$$

where $E_{cost}\left(l\right)$ represents the energy consumption of node l in the routing, which primarily consists of the energy consumed by receiving data from the upper-level CH node and forwarding data. Its calculation formula is given in Equation (16).

(16) $$E_{cost}\left(l\right)=E_{elec}\times p\left(l\right)+E_{fs}\times p\left(l\right)\times {dis\left(l,CH_{next}\right)}^{2}+E_{elec}\times p_{uper}\left(l\right)$$

where $E_{elec}$ and $E_{fs}$ are parameters in the first-order radio model, p(l) denotes the total amount of data that node l needs to forward, $p_{uper}\left(l\right)$ denotes the total amount of data that l receives from its upper-level CH, and dis(l,CH_next) represents the distance from l to the next-hop destination CH. The calculation formula for dis(l,CH_next) is given in Equation (17).

(17) $$dis\left(l,\mathrm{CH\_next}\right)=\sqrt[{2}]{{\left(l_{\_x}-{\mathrm{CH\_next}}_{\mathrm{\_x}}\right)}^{2}+{\left(l_{\_y}-{\mathrm{CH\_next}}_{\mathrm{\_y}}\right)}^{2}}$$

In this study, the maximum cluster competition radius is set to $R_{max}$ = 100 m, and a non-uniform clustering mechanism is adopted. CHs are linearly and orderly distributed along the longitudinal direction of the narrow monitoring area. Moreover, the width of the monitoring region is only 50 m, and the maximum lateral distance difference between adjacent cluster heads does not exceed 50 m. Considering the constraints imposed by the competition radius, the actual Euclidean distance between adjacent cluster heads is naturally restricted within a reasonable range, thereby avoiding excessively long inter-cluster single-hop transmissions from a topological perspective.

- Energy Consumption Variance Function

The variance of energy consumption among CHs helps evaluate the distribution of energy consumption across nodes. A smaller energy consumption variance indicates that the energy consumption of each node in the network is relatively balanced, which helps maintain the overall equilibrium of the network. A larger energy consumption variance may imply that the energy consumption of some nodes deviates from the mean, requiring further adjustments to improve the overall network performance. By considering the energy consumption variance, the algorithm can be encouraged to allocate CHs more evenly in routing decisions, avoiding excessive load in certain local areas. The specific calculation method of the energy consumption variance function is given in Equation (18).

(18) $$f_{2}=\frac{1}{\mathrm{N\_CH}}\sum_{l\in CH}{\left(E_{cost}\left(l\right)-E_{cost\_av}\right)}^{2}$$

where N_CH denotes the number of CHs in the network, $E_{cost\_av}$ represents the average energy consumption of all current CHs, and CH represents the set of CHs in the current network. The average energy consumption of CHs, $E_{cost\_av}$, is expressed as Equation (19).

(19) $$E_{cost\_av}=\frac{1}{\mathrm{N\_CH}}\sum_{l\in CH}E_{cost}\left(l\right)$$

The above two functions are combined into the final objective function by means of linear weighting, as shown in Equation (20).

(20) $$f=w_{1}\times f_{1}+w_{2}\times f_{2}$$

where $w_{1}$ and $w_{2}$ represent the weights of the total energy consumption factor and the energy consumption balance factor in the final objective function, respectively. Most multi-hop routing algorithms assign weights to multiple constructed objective functions based on expert knowledge, which suffers from strong subjectivity and requires extensive professional expertise. To better adapt to changes in network status, the ISSAMC algorithm dynamically assigns weights to each component of the objective function through a nonlinear function based on the network residual energy threshold, whose specific expression is shown in Equation (21).

(21) $$f\left(E_{res}\right)=\frac{1}{1+e^{-\mu \left(E_{res}-\varphi \times E_{total}\right)}}$$

where $E_{res}$ denotes the residual energy of the current network, $E_{total}$ represents the total network energy, and φ and μ are hyper parameters used for function adjustment. When the residual energy in the network is sufficient, more emphasis should be placed on the energy consumption balance of network nodes, so the weight of the energy consumption balance factor ought to be larger. When the residual energy of the network is low, priority should be given to network lifetime, and thus a greater weight should be assigned to the total energy consumption factor. In summary, the weight calculation formulas of the objective function in this paper are presented in Equations (22) and (23).

(22) $$w_{2}=\frac{1}{1+e^{-\mu \left(E_{res}-\varphi \times E_{total}\right)}}$$

(23) $$w_{1}=(1-w_{2})$$

### 4.4. Data Transmission Phase

After the BS determines the CHs and inter-cluster data transmission paths, it broadcasts CH information (CH_Msg) and inter-cluster transmission path information (PATH_Msg) to all network nodes. The CH information (CH_Msg) mainly contains the set of CH IDs in the current network round, which is used to designate CH nodes and specify the cluster affiliation of ordinary nodes. The inter-cluster transmission path information (PATH_Msg) primarily includes the multi-hop paths among CHs, aiming to define the next-hop address for each CH.

**Figure 6:**
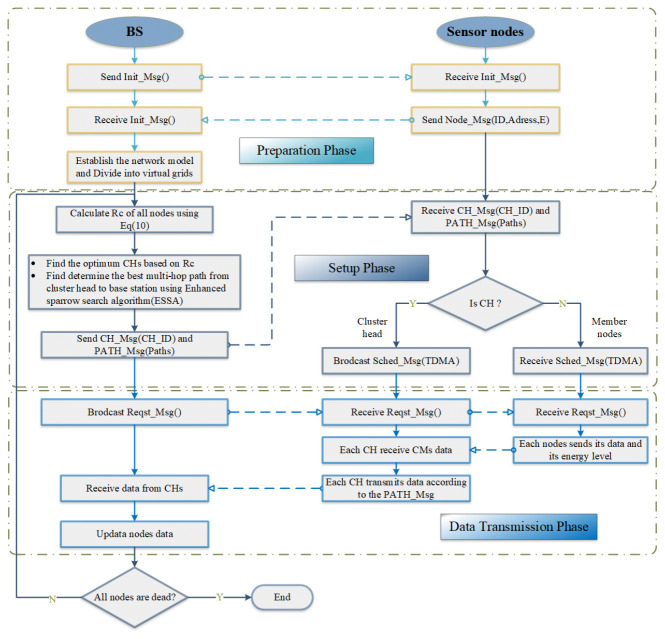
Flowchart of ISSAMC.

Once the roles of CH nodes in the network are confirmed, each CH generates a Time Division Multiple Access (TDMA) schedule and broadcasts the scheduling information within its cluster. Upon receiving the Request_Msg from the BS, each sensor node transmits its sensed data and status information to the corresponding CH during its allocated time slot. After collecting the sensing data from member nodes, the CH forwards the data to the BS along the established multi-hop paths. When the BS receives data from all nodes, it automatically updates the stored node information and initiates a new round. The detailed flowchart of the ISSAMC algorithm is illustrated in Figure 6.

The pseudo-code of ISSAMC is shown in Algorithm 1. First, the network is partitioned, calculating the number of nodes in each region and completing the network initialization (lines 1–2). Then, the BS determines the CHs by a competition radius-based cluster formation [16] algorithm (lines 5–10). Next, the BS finds the optimal inter-cluster multi-hop routes by a modified sparrow search algorithm (lines 11–12). When the BS determines the CHs and inter-cluster transmission paths, it broadcasts them to the entire network. When the BS determines the CHs and inter-cluster transmission paths, it broadcasts them to the entire network. Subsequently, common nodes join the corresponding CH. The CH node creates a Generation Time Division Multiple Access (TDMA) scheduling and performs intra-cluster broadcasting (lines 13–14). During the data transmission phase, the cluster member nodes transmit the data to the CH via single hop and the CH transmits the data to the BS according to the optimal multi-hop routing (lines 15–16). Finally, the BS updates the information of the nodes based on the returned data and records the network information for this round, including the remaining energy of the network and the survival of the nodes (line 17).
**Algorithm 1** Pseudo-code of the proposed protocolInput: $N,{S,E}_{0},\alpha ,\beta ,R_{max}$
Output: Network operation information.1. Start:2.   Network initialize3.   while $E_{rs}>0$ do4.       for each subzone do5.             Select some nodes with high residual energy as CCH.6.             for each CCH do7.                   Calculate $R_{c}$ according Equation (10)8.             end9.           Using unequal clustering algorithm determine the final CH.10.    end11.        Calculate $w_{1}$, $w_{2}$ by Equations (22) and (23).12.        Using ESSA find the best multi-hop route.13.        The ON nodes in each region join the nearest CH.14.        CH generates TDMA scheduling.15.        ON transmits the data to its own CH.16.        CH transmit data to the BS following the best multi-hop route.17.        Statistical network operation information.18.   end19.   Return Network operation information20. end

## 5. Simulation and Analysis

To evaluate the performance of ISSAMC, extensive simulation experiments were conducted in MATLAB 2022. Meanwhile, it was compared with MH-LEACH [14], EBPSO [31], GAECH [28], and BEBMCR [34] under the same experimental environment to verify that ISSAMC is more suitable for long and narrow monitoring scenarios. The specific experimental parameters are presented in Table 1. In the simulation, the fixed data packet includes both the effective sensing payload and MAC-layer protocol overhead, which is consistent with the typical payload length of the IEEE 802.15.4 standard. The sampling rate of each node is set to 1 Hz, and the data reporting period is consistent with the routing update period. In each round, each node reports one data packet, which meets the common sampling requirements of structural health monitoring. The initial node energy used in this study is a normalized simulation baseline value rather than the actual battery capacity of real IoT nodes.

### 5.1. Determination of Hyperparameter φ

**Figure 7:**
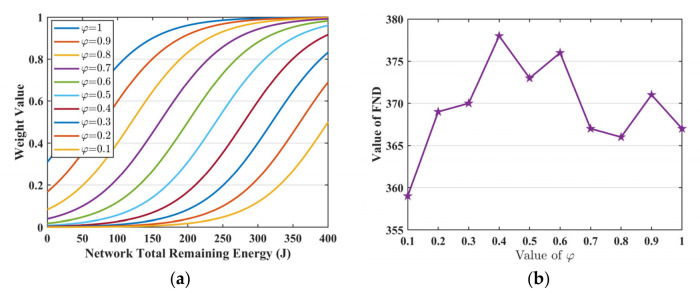
Influence of different φ values. (**a**) Influence of different φ values on the weight $\omega_{2}$ of the fitness function. (**b**) Influence of different φ values on FND.

Before conducting a comparative analysis on various performance indicators of the ISSAMC algorithm, the influence of the hyperparameter φ on ISSAMC is first investigated. Firstly, 200 nodes are deployed in a strip area of 1000m × 50 m, and the entire network is equally divided into 10 sub-networks at equal intervals. According to Equation (5), the number of nodes in each sub-network is obtained as [7,9,12,16,19,22,24,27,31,33]. Figure 7a illustrates the curves of the control function under different values of φ. Figure 7b presents the First Node Death (FND) time and Last Node Death (LND) time of ISSAMC with varying φ values. It can be observed from Figure 7b that ISSAMC achieves a significantly better FND performance when φ is set to 0.4 compared with other settings. Combined with the analysis of the nonlinear control function at φ = 0.4 in Figure 7a, ISSAMC prioritizes balancing the energy consumption among network nodes when the residual network energy exceeds 70%. When the residual network energy is lower than 70%, ISSAMC focuses more on energy saving and assigns a larger weight to node energy consumption control. Consequently, the value of φ is determined to be 0.4.

### 5.2. CH Distribution and Multi-Hop Paths

**Figure 8:**
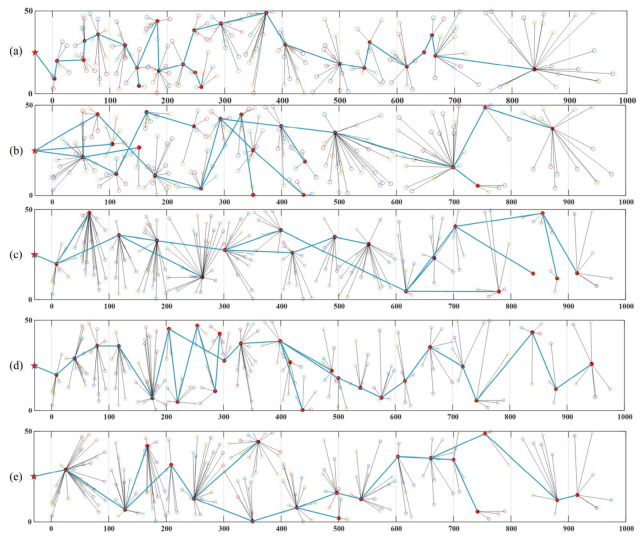
Inter-cluster transmission paths of the five algorithms. (**a**) MH-LEACH; (**b**) GAECH; (**c**) BEBMCR; (**d**) EBPSO; (**e**) ISSAMC.

To intuitively present the CH distribution and data transmission paths in the network and compare the performance differences of different routing algorithms in long and narrow monitored WSNs, Figure 8 illustrates the CH distribution and multi-hop paths of the five algorithms.

Figure 8a shows the inter-cluster multi-hop paths of MH-LEACH. Since MH-LEACH selects CHs randomly and each CH only forwards data to the nearest CH, the CHs close to the BS exhaust energy rapidly and die prematurely, resulting in a short stability period and poor robustness of this algorithm.

Figure 8b depicts the inter-cluster multi-hop paths of GAECH. This algorithm adopts the genetic algorithm for multi-hop path optimization. However, the genetic algorithm exhibits prominent fluctuation during the solution process, leading to a large difference in the transmission distance of CHs, which further causes significant disparity in node energy consumption.

Figure 8c presents the inter-cluster multi-hop routing of BEBMCR. This algorithm controls the cluster scale, thus achieving relatively uniform cluster size. Nevertheless, CHs in this algorithm bear long data transmission distances and consume excessive energy, making it ineffective in energy conservation.

Figure 8d provides the inter-cluster multi-hop routing of EBPSO. The multi-hop routes constructed by EBPSO maintain small fluctuations in CH transmission distance, whereas the CHs near the BS are prone to premature death due to excessive communication load.

Figure 8e demonstrates the inter-cluster multi-hop routing of ISSAMC. In the multi-hop routes established by ISSAMC, the data transmission distance of each CH is approximately equal. Accordingly, ISSAMC achieves remarkably better energy consumption balance among nodes than other algorithms, which can effectively prolong the stability period and lifetime of WSNs. Compared with other algorithms, it is more applicable to long and narrow monitoring areas.

### 5.3. Stability Period and Network Lifetime

The network stability period refers to the time interval from network initialization to the FND. A longer stability period indicates more balanced energy consumption among network nodes. WSN nodes in long and narrow monitoring scenarios are usually deployed in harsh environments with difficult maintenance. Therefore, it is necessary to design routing algorithms with a long stability period to improve the practical availability of the network.

**Figure 9:**
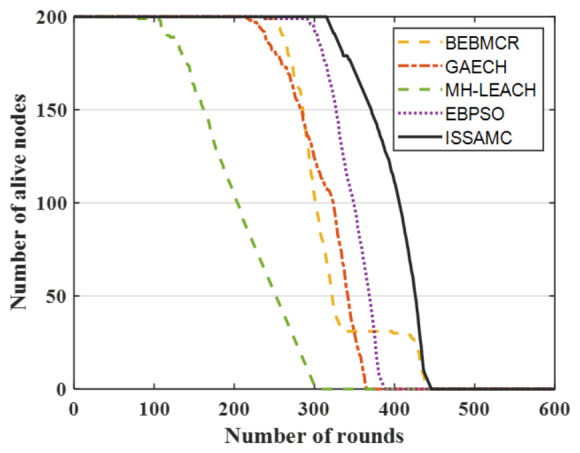
The lifecycle of the network.

**Figure 10:**
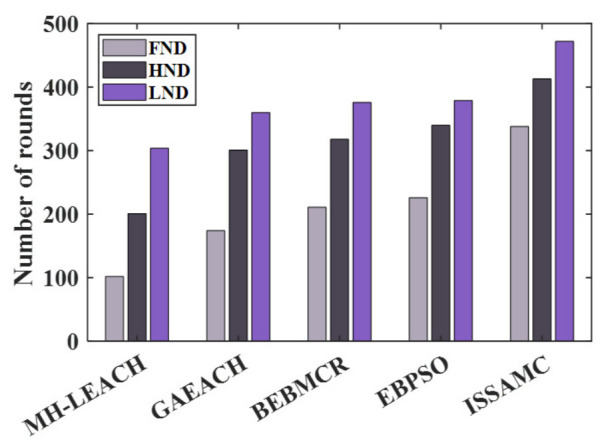
FND, HND and LND.

Figure 9 illustrates the node survival status of the five algorithms, and the corresponding FND, Half Node Death (HND), and LND are shown in Figure 10. The FND of ISSAMC reaches 338 rounds, while those of EBPSO, BEBMCR, GAECH and MH-LEACH are 226 rounds, 211 rounds, 174 rounds and 103 rounds, respectively. Compared with MH-LEACH, GAECH, BEBMCR and EBPSO, the stability period of ISSAMC is increased by 231%, 94%, 60% and 49.5%, respectively. The LND of ISSAMC is 472 rounds, which is 55%, 31%, 25.5% and 24% higher than that of MH-LEACH, GAECH, BEBMCR and EBPSO.

When searching for the optimal multi-hop path, ISSAMC takes the minimization of total network energy consumption and node energy consumption variance as the optimization objectives, which enables ISSAMC to effectively extend the network stability period and network lifetime.

In this study, one simulation round corresponds to a complete cycle of data acquisition, intra-cluster aggregation, and multi-hop data reporting, which can be directly mapped to physical time according to the actual sampling and reporting frequency of the target scenario. For example, under a conventional sampling strategy for bridge structural health monitoring, one round may correspond to a physical period of one minute, and the total network lifetime can therefore be converted into actual operating time. According to the energy consumption characteristics of low-power sensing nodes [46], the power consumption of nodes in sleep mode is two to three orders of magnitude lower than that during communication. Under a conventional duty-cycle working mode, the influence of sleep energy consumption on the total lifetime is much smaller than the difference in communication energy consumption. Therefore, the round-based performance comparison conclusions are robust and will not change the relative ranking of different algorithms due to the introduction of sleep duration.

**Figure 11:**
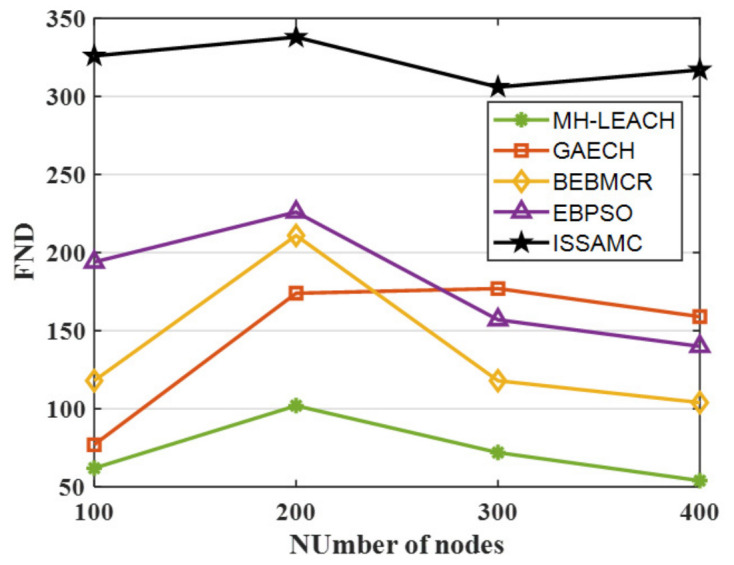
FND of the five algorithms under different node densities.

To verify the performance stability of ISSAMC under different node densities, the total number of network nodes is set to 100, 200, 300 and 400, respectively. As shown in Figure 11, MH-LEACH, GAECH, EBPSO and BEBMCR achieve the longest network stability period when the number of nodes is 200. Nevertheless, with the increase in node number, especially when the number exceeds 200, nodes near the BS need to forward more data, which shortens the node survival time and reduces network stability. On the contrary, ISSAMC presents a remarkably longer network stability period than other algorithms under different node densities. This indicates that ISSAMC can effectively balance node energy consumption and improve network stability.

### 5.4. Energy Consumption

**Figure 12:**
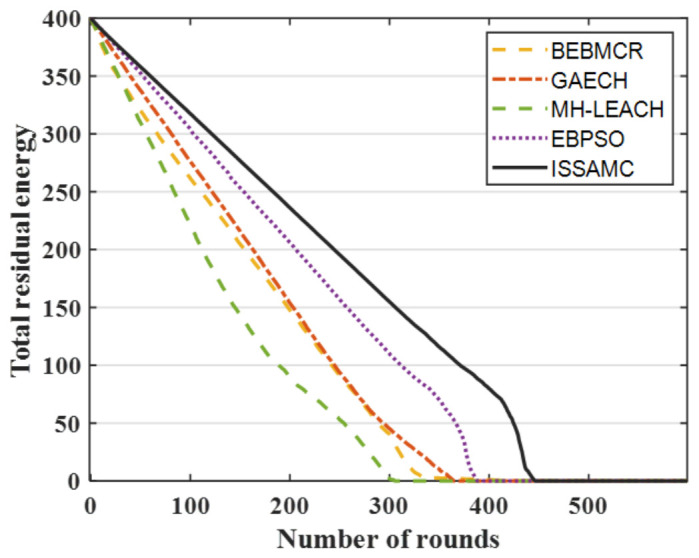
Network residual energy.

Minimizing node energy consumption is one of the core objectives of WSN algorithm design. By adjusting cluster scale and optimizing inter-cluster multi-hop paths, the proposed method successfully reduces node energy consumption and remarkably prolongs the overall network lifetime. Figure 12 clearly depicts the variation of network residual energy with simulation rounds under different algorithms. Notably, the ISSAMC algorithm maintains lower energy consumption than the other four algorithms across all rounds. This advantage benefits from the targeted improvement of the sparrow search algorithm, which can determine appropriate inter-cluster transmission paths and effectively reduce node energy consumption.

**Figure 13:**
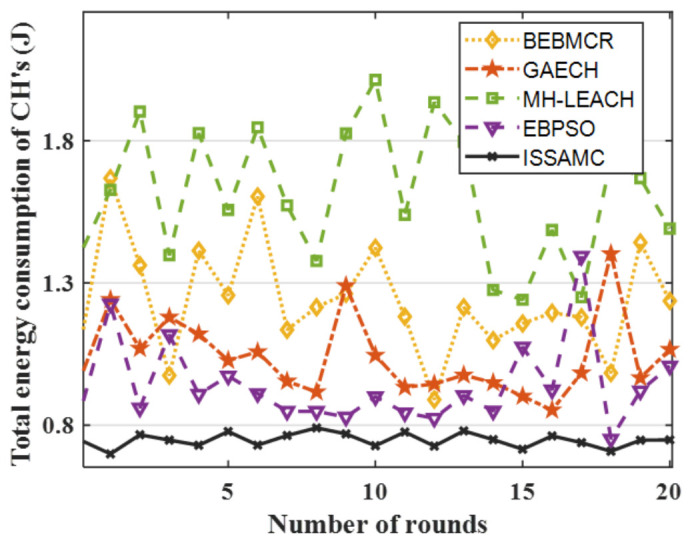
Total energy consumption of the CHs.

A prominent characteristic of long and narrow monitored WSNs lies in the large span of the monitoring area, whereby CHs need to transmit data to the BS in a multi-hop manner. CHs undertake the task of forwarding data collected by intra-cluster member nodes, resulting in much higher energy consumption than ordinary nodes. In this paper, 20 simulation rounds are randomly selected to statistically analyze the total energy consumption of all CHs in each round for different algorithms. The corresponding results are illustrated in Figure 13. It can be observed that the total CH energy consumption of ISSAMC is obviously lower than that of the other four algorithms, with minor fluctuation. This is because the minimum total energy consumption of CHs is taken as one of the optimization objective functions when solving inter-cluster multi-hop paths, thereby constructing more energy-efficient multi-hop routes.

### 5.5. Energy Consumption Balance

WSNs deployed in long and narrow monitoring scenarios suffer from severe unbalanced node energy consumption. The Balance Degree of Energy Dissipation (BEED) is one of the widely adopted metrics for evaluating the energy balance performance of algorithms, and its calculation formula is defined as BEED=(LND−FND)/LND. A smaller BEED value indicates a better performance in balancing energy consumption. Table 2 lists the BEED values of different algorithms. The BEED of the proposed ISSAMC is 0.284, which is obviously lower than those of the other four algorithms. This further verifies the superior performance of ISSAMC in terms of energy consumption balance.

**Figure 14:**
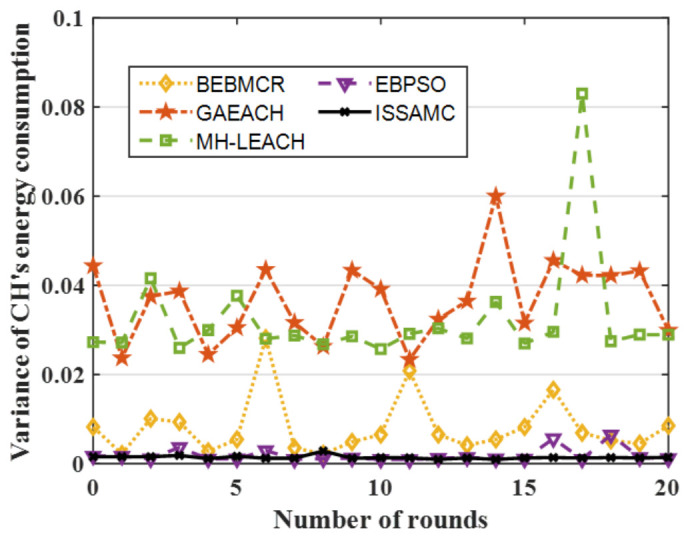
Variance of CH energy consumption.

Node energy consumption variance serves as an intuitive metric for evaluating the energy balance among nodes. Especially in multi-hop routing algorithms, CHs consume significantly more energy than ordinary nodes; accordingly, the energy balance among CHs can well reflect the overall energy distribution of network nodes. In Figure 14, the energy consumption variance of CHs for different algorithms is statistically analyzed over 20 randomly selected simulation rounds.

The energy consumption variance of CHs under ISSAMC and EBPSO is much lower than that of the other algorithms, while the proposed ISSAMC exhibits smaller fluctuation and higher stability in variance. As variants of the classic LEACH algorithm, GAECH and MH-LEACH adopt a random CH selection strategy without controlling cluster scale, thus yielding the largest variance. BEBMCR and EBPSO adopt the idea of unequal clustering to regulate cluster size and employ intelligent optimization algorithms to optimize inter-cluster data transmission paths, resulting in a lower variance than GAECH and MH-LEACH. Nevertheless, the genetic algorithm suffers from severe fluctuations during the optimization process, leading to considerable variance volatility of BEBMCR.

For ISSAMC, clusters with different sizes are first formed by setting diverse competition radii. The competition radius of each node is calculated by comprehensively considering node location and residual energy, enabling the CH distribution to better adapt to long and narrow monitoring scenarios. Furthermore, the energy consumption variance among CHs is taken as one of the optimization objectives, which further reduces the variance of CH energy consumption.

In summary, ISSAMC possesses a stronger capability in balancing node energy consumption compared with other algorithms, making it more suitable for WSNs in long and narrow monitoring scenarios.

### 5.6. Network Data Transmission Volume

**Figure 15:**
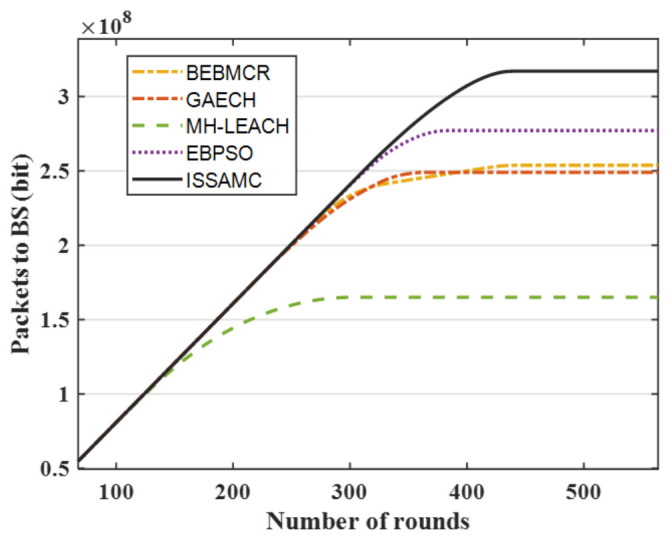
Data volume received by the BS.

The primary task of WSNs is to conduct automatic real-time monitoring of the target area and transmit the monitored data to the BS in a timely manner. Figure 15 presents the total amount of data received by the BS under the five algorithms. It can be observed that the total data volume received by the BS in the ISSAMC scheme is significantly higher than that of the other algorithms. This is because the multi-hop paths constructed by the proposed algorithm are more energy-efficient, which extends the network monitoring duration for the sensed area to the greatest extent. Consequently, ISSAMC can still continue to receive data when other algorithms terminate data reception.

## 6. Discussion and Conclusions

This section first summarizes the main research work and performance conclusions of this study. On this basis, it further discusses the model boundaries, engineering applicability, and simulation limitations of the study, and provides an outlook on future research directions.

### 6.1. Main Conclusions

To address the energy hole problem and limited network lifetime of wireless sensor networks in long and narrow strip-shaped monitoring scenarios, this study proposes a non-uniform clustering multi-hop routing algorithm based on an improved sparrow search algorithm. The algorithm achieves non-uniform clustering by optimizing the node competition radius, which alleviates the forwarding load pressure on cluster heads close to the base station. Meanwhile, Sobol sequence-based population initialization and an adaptive optimization strategy are introduced to enhance the path optimization performance of the sparrow search algorithm, supporting the construction of energy-balanced inter-cluster multi-hop transmission paths. Simulation results under a consistent benchmark demonstrate that compared with similar clustering routing algorithms, the proposed algorithm effectively extends the network stability period and overall lifetime and improves the balance of node energy consumption. All performance conclusions are drawn within an idealized simulation framework, which can provide theoretical reference and algorithmic support for WSN routing design in health monitoring scenarios of long and narrow infrastructures such as railways and bridges. The practical engineering applicability of the method requires further verification against real deployment environments.

### 6.2. Research Limitations and Applicability

This study evaluates the proposed algorithm through simulation experiments, and its applicability should be understood within the following boundaries:

(1) The simulation adopts the first-order LEACH radio energy model, which is widely used in WSN studies but relies on simplified assumptions. Specifically, the model assumes static node deployment, stable wireless channels, no burst interference, no packet retransmission, no clock synchronization error, and ignores standby energy consumption and environmental noise. These assumptions help ensure controlled and fair comparisons among different algorithms, but they may also cause deviations from real wireless environments. Therefore, the actual performance of the proposed algorithm under channel fading, packet loss, retransmission, duty-cycling mechanisms, synchronization overhead, and hardware-specific energy parameters still requires further engineering validation.
(2) The proposed non-uniform clustering and multi-hop routing strategy is mainly designed for long and narrow monitoring scenarios. In practical deployment, additional modules such as localization error correction, adaptive link-quality adjustment, and fault-tolerant node switching are needed for engineering adaptation. This study focuses on routing optimization at the terminal multi-hop aggregation layer, which is different from LPWAN star networks or hybrid gateway architectures. These technologies are complementary in practical infrastructure monitoring systems rather than mutually exclusive. Therefore, the conclusions of this paper are limited to comparisons with peer clustering routing schemes under the same simulation framework.
(3) The simulation results represent the algorithm’s relative performance under ideal and controllable conditions. In real WSN or IoT systems, factors such as multipath fading, co-channel interference, environmental variations, and hardware heterogeneity may increase node energy consumption and reduce network lifetime. Although the proposed algorithm shows advantages over similar clustering routing schemes in simulation, its quantitative performance in real deployments still needs to be calibrated and verified using specific hardware platforms and application scenarios.

### 6.3. Future Work

Future research will narrow the simulation-engineering gap via field hardware tests and more realistic channel fading and node failure models. The integrated optimization of energy harvesting and routing for elongated scenarios will also be explored to enhance sustainable network operation.

## Figures and Tables

**Table 1:** Simulation parameters.

Categories	Parameters	Value
Network parameters	Network area (m)	1000 × 50
	Total number of nodes n	200
	Position of BS	(−30, 25)
	The initial energy of nodes (J)	2
	$R_{max};R_{min}$ (m)	100; 20
	Packet size (bit)	4000
	$E_{elec}\left(nJ/bit\right)$	50
	$E_{fs}\left(pJ/bit/m^{2}\right)$	10
	$E_{mp}\left(pJ/bit/m^{4}\right)$	0.0013
SSA	Number of populations	30
	Number of discovers PD	9
	Number of scouters SD	6
	Alarm threshold ST	0.8
	Transverse crossover probability $P_{T}$	0.8
	Longitudinal crossover probability $P_{L}$	0.2

**Table 2:** The BEED of five algorithms.

Protocol	FND	LND	BEED
MH-LEACH	102	305	0.665
BEBMCR	211	376	0.438
GAECH	174	360	0.517
EBPSO	226	379	0.404
ISSAMC	338	472	0.284

## Data Availability

Data are contained within the article.

## References

[1] Yang J., Huo J., Cao F., Mu C. (2026). Multi-objective Deployment Optimization and Final Solution Decision for Heterogeneous WSN Nodes in Elongated Structure Spaces. IEEE Sens. J..

[2] Najjar S., David M., Derigent W., Zouinkhi A. (2025). Dynamic reconfiguration of wireless sensor networks: A survey. Comput. Netw..

[3] Cao B., Zhang L., Han M., Li M., Xu B., Zeng Q., Bao G., Yao Z. (2025). UWB sensors optimization deployment for narrow and long space environments. IEEE Access.

[4] Chu A., Zhang T., Wang C. (2026). LEACH Protocol Evolution in WSN: A Review of Energy Consumption Optimization and Security Reinforcement. Sensors.

[5] Jukuntla A. (2025). Enhancing energy efficiency in WSNs using fuzzy logic-based LEACH protocol with rendezvous nodes and mobile sink. Measurement.

[6] Zhao J., Liu B., Zhang L. (2025). Quantum adaptive clonal genetic algorithm for low-energy clustering in agricultural WSNs. Sci. Rep..

[7] Manasa B., RamaKrishna D. (2025). Energy-efficient PSO-QLR routing in wireless sensor networks. AEU-Int. J. Electron. Commun..

[8] Wang L., Luo Y., Yan H. (2024). Optimization analysis of node energy consumption in wireless sensor networks based on improved ant colony algorithm. Sustain. Energy Technol. Assess..

[9] Xue J., Shen B. (2020). A novel swarm intelligence optimization approach: Sparrow search algorithm. Syst. Sci. Control Eng..

[10] Gharehchopogh F.S., Namazi M., Ebrahimi L., Abdollahzadeh B. (2023). Advances in sparrow search algorithm: A comprehensive survey. Arch. Comput. Methods Eng..

[11] Paterova T., Prauzek M., Konecny J., Ozana S., Zmij P., Stankus M., Weise D., Pierer A. (2021). Environment-monitoring IoT devices powered by a TEG which converts thermal flux between air and near-surface soil into electrical energy. Sensors.

[12] Singh S.K., Kumar P., Singh J.P. (2017). A survey on successors of LEACH protocol. IEEE Access.

[13] Khedr A.M., Aziz A., Osamy W. (2021). Successors of PEGASIS protocol: A comprehensive survey. Comput. Sci. Rev..

[14] Neto J.H.B., Rego A., Cardoso A.R., Celestino J. MH-LEACH: A distributed algorithm for multi-hop communication in wireless sensor networks. Proceedings of the ICN.

[15] Al-Sodairi S., Ouni R. (2018). Reliable and energy-efficient multi-hop LEACH-based clustering protocol for wireless sensor networks. Sustain. Comput. Inform. Syst..

[16] Jin R., Fan X., Sun T. (2021). Centralized multi-hop routing based on multi-start minimum spanning forest algorithm in the wireless sensor networks. Sensors.

[17] Siamantas G., Rountos D., Kandris D. (2025). Energy saving in wireless sensor networks via LEACH-based, energy-efficient routing protocols. J. Low Power Electron. Appl..

[18] Elkamel R., Messouadi A., Cherif A. (2019). Extending the lifetime of wireless sensor networks through mitigating the hot spot problem. J. Parallel Distrib. Comput..

[19] Li C., Chen G., Ye M., Wu J. (2007). An Uneven Cluster-Based Routing Protocol for Wireless Sensor Networks. Chin. J. Comput..

[20] He B., Li G. (2014). PUAR: Performance and usage aware routing algorithm for long and linear wireless sensor networks. Int. J. Distrib. Sens. Netw..

[21] Nivedhitha V., Saminathan A.G., Thirumurugan P. (2020). DMEERP: A dynamic multi-hop energy efficient routing protocol for WSN. Microprocess. Microsyst..

[22] Jing C., Zhu X., Liu X. (2023). Performance analysis model and deterministic routing decision algorithm for broadband real-time services in wireless multi-hop networks. IEEE Trans. Veh. Technol..

[23] Gupta M., Singh Aulakh N., Kaur Aulakh I. (2022). A game theory-based clustering and multi-hop routing scheme in wireless sensor networks for energy minimization. Int. J. Commun. Syst..

[24] Amutha J., Sharma S., Sharma S.K. (2022). An energy efficient cluster based hybrid optimization algorithm with static sink and mobile sink node for wireless sensor networks. Expert Syst. Appl..

[25] Singh A., Sharma S., Singh J. (2021). Nature-inspired algorithms for wireless sensor networks: A comprehensive survey. Comput. Sci. Rev..

[26] Bari A., Wazed S., Jaekel A., Bandyopadhyay S. (2009). A genetic algorithm based approach for energy efficient routing in two-tiered sensor networks. Ad. Hoc Netw..

[27] Zhang B., Cao J. (2010). Uneven Clustering Routing Algorithm for Wireless Sensor Networks Based on Ant Colony Optimization. J. Xi’an Jiaotong Univ..

[28] Baranidharan B., Santhi B. (2015). GAECH: Genetic algorithm based energy efficient clustering hierarchy in wireless sensor networks. J. Sens..

[29] Al-Shalabi M., Anbar M., Wan T.-C., Alqattan Z. (2019). Energy efficient multi-hop path in wireless sensor networks using an enhanced genetic algorithm. Inf. Sci..

[30] Vinitha A., Rukmini M.S.S., Sunehra D. (2020). Energy-efficient multihop routing in WSN using the hybrid optimization algorithm. Int. J. Commun. Syst..

[31] Xiuwu Y., Zixiang Z., Wei P., Yong L. (2023). A novel multi-hop clustering routing algorithm based on particle swarm optimization for wireless sensors networks. Wirel. Pers. Commun..

[32] Rani K.P., Sreedevi P., Poornima E., Sri T.S. (2023). FTOR-Mod PSO: A fault tolerance and an optimal relay node selection algorithm for wireless sensor networks using modified PSO. Knowl. Based Syst..

[33] Kaedi M., Bohlooli A., Pakrooh R. (2022). Simultaneous optimization of cluster head selection and inter-cluster routing in wireless sensor networks using a 2-level genetic algorithm. Appl. Soft Comput..

[34] Gong Y., Cao J., Han C., Liu Y. (2022). Energy Balance Multihop Clustering Routing Protocol for Large-Scale Water Quality Monitoring. Wirel. Commun. Mob. Comput..

[35] Luo X., Zhang C., Bai L. (2023). A fixed clustering protocol based on random relay strategy for EHWSN. Digit. Commun. Netw..

[36] Yang X., Zhu W., Li J., Zeng X., Qiu Y. (2026). An Adaptive Clustering Routing Algorithm with High Survivability Based on Dynamic Hierarchical Head Backup for Underwater Optical-Acoustic Hybrid Wireless Sensor Networks. IEEE Sens. J..

[37] Salman N., Rasool I., Kemp A.H. Overview of the IEEE 802.15. 4 standards family for low rate wireless personal area networks. Proceedings of the 2010 7th International Symposium on Wireless Communication Systems.

[38] Meng J., Wang J., Li D., Liu Y., Chen X. (2023). Efficient Routing Protocol for Linear Wireless Sensor Networks along the Railway. China Railw. Sci..

[39] Luo T., Xie J., Zhang B., Zhang Y., Li C., Zhou J. (2024). An improved levy chaotic particle swarm optimization algorithm for energy-efficient cluster routing scheme in industrial wireless sensor networks. Expert Syst. Appl..

[40] Kaviarasan S., Srinivasan R. (2024). Developing a novel energy efficient routing protocol in WSN using adaptive remora optimization algorithm. Expert Syst. Appl..

[41] Daneshvar S.M.M.H., Mazinani S.M. (2023). On the best fitness function for the WSN lifetime maximization: A solution based on a modified salp swarm algorithm for centralized clustering and routing. IEEE Trans. Netw. Serv. Manag..

[42] Wang Z., Peng Q., Rao W., Li D. (2025). An improved sparrow search algorithm with multi-strategy integration. Sci. Rep..

[43] Zhang Z., Han Y. (2022). Discrete sparrow search algorithm for symmetric traveling salesman problem. Appl. Soft Comput..

[44] Sirsant S., Reddy M.J. (2022). Improved MOSADE algorithm incorporating Sobol sequences for multi-objective design of Water Distribution Networks. Appl. Soft Comput..

[45] Yue Y., Cao L., Lu D., Hu Z., Xu M., Wang S., Li B., Ding H. (2023). Review and empirical analysis of sparrow search algorithm. Artif. Intell. Rev..

[46] Prauzek M., Konecny J., Borova M., Janosova K., Hlavica J., Musilek P. (2018). Energy harvesting sources, storage devices and system topologies for environmental wireless sensor networks: A review. Sensors.

